# Supplementary material for: Functional Crosstalk between PCSK9 Internalization and Pro-Inflammatory Activation in Human Macrophages: Role of Reactive Oxygen Species Release
Source: Int J Mol Sci. 2022 Aug 14;23(16):9114. doi: 10.3390/ijms23169114 (PMC9409451; doi:10.3390/ijms23169114)
Supplement: Supplementary file 1 [file ijms-23-09114-s001.zip › ijms-1809689-supplementary.pdf]

Supplementary tables:

Table S1: Primary and secondary antibodies used in Western Blot assays.

**Antibody**	**Supplier**	**Reference**
Anti-PCSK9	Abcam	Ab181142
Anti-TLR4	Santa Cruz Biotech.	Sc-293072
Anti-P-p38 (Thr180/Tyr182)	Cell Signaling	#4511
Anti-p38	Cell Signaling	#8690
Anti-vinculin	Cell signaling	#18799
Anti-caspase-1 p10	Cell Signaling	#3866 AF4022
Anti-cleaved caspase-3	Cell Signaling	#9664
Anti-IκB-α	Cell Signaling	#9242
Anti-P-IκB-α (Ser32)	Cell Signaling	#2859
Anti-P-p65 (Ser536)	Cell Signaling	#3033
Anti-p65	Cell Signaling	#8242
Anti-β-actin	Sigma	A5441
Anti-rabbit IgG	Bio-Rad	#1706515
Anti-mouse IgG	Sigma	A-4416
Anti-goat IgG	Sigma	A-5420

Table S2. Specific primer sequences used in qRT-PCR assay (sequences; 5′–3′).

Gen	Forward	Reverse
Human primer sequences		
ABCA1	ACCCACCCTATGAACAACATGA	GAGTCGGGTAACGGAAACAGG
ABCG1	CGTGCGCTTTGTGCTGTTT	CCACTGTAGGTACGTGGGGAT
CD36	CTTTGGCTTAATGAGACTGGGAC	GCAACAAACATCACCACACCA
CYBB	ACCGGGTTTATGATATTCCACCT	GATTTCGACAGACTGGCAAGA
FASN	AAGGACCTGTCTAGGTTTGATGC	TGGCTTCATAGGTGACTTCCA
HMGCR	TGATTGACCTTTCCAGAGCAAG	CTAAAATTCGCATTCCACGAGC
IL1B	TCGAGGCACAAGGCACAACAGG	GCCATGGCTGCTTCAGACACTTGA
ITGAM	GCCTTGACCTTATGTCATGGG	CCTGTGCTGTAGTCGCACT
LDLR	TCTGCAACATGGCTAGAGACT	TCCAAGCATTCGTTGGTCCC
NLRP3	GATCTTCGCTGCCATCAACAG	CGTGCATTATCTGAACCCCAC
PCSK9	GGACTCCTCTGTCTTTGCCC	TCCCGGTGGTCACTCTGTAT
SREBP1	CGCAACCATCTTGGCAACAGT	CGCTTCTCAATGGCGTTGT
TNF	GAGGCCAAGCCCTGGTATG	CGGGCCGATTGATCTCAGC
36B4	GGATTACACCTTCCCACTTGC	GCCACAAAGGCAGATGGATCA
Mouse primer sequences		
Il1b	AGAAGCTGTGGCAGCTACCTG	GGAAAAGAAGGTGCTCATGTCC
Pcsk9	GCCCATCGGGAGATTGAGG	TTCCCTTGACAGTTGAGCACA
Tnf	CATCTTCTCAAAATTCGAGTGACAA	TGGGAGTAGACAAGGTACAACCC
36b4	ACTGGTCTAGGACCCGAGAAG	TCCCACCTTGTCTCCAGTCT

Supplementary figures:

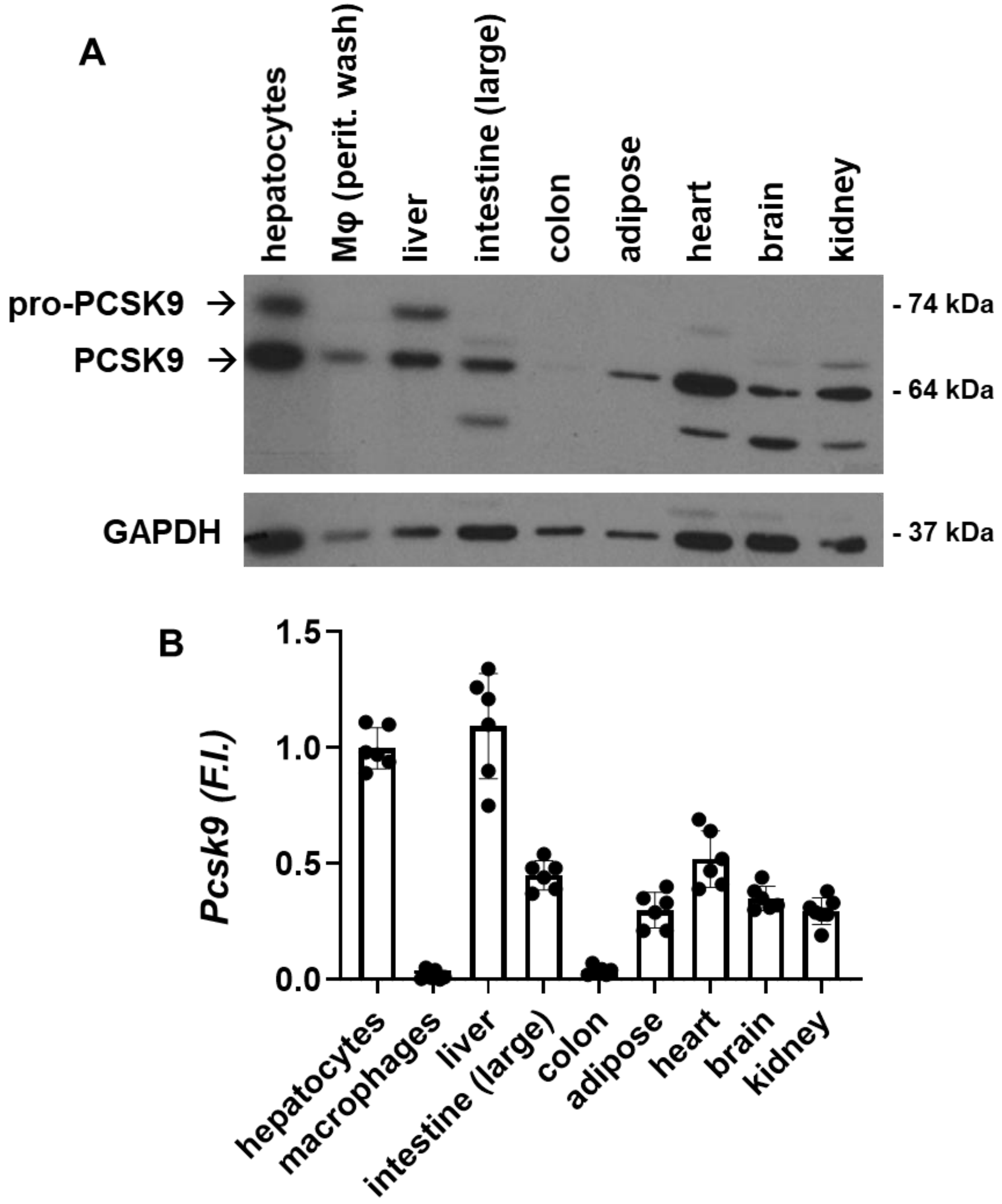

Figure **S1*. Intracellular content of PCSK9 and mRNA levels of Pcsk9 in murine tissues***. (**A**) Quantification of PCSK9 in different murine tissues. Mice were fed ad libitum and tissues and cells were prepared, homogenized and the protein levels were determined by Western blot. (**B**) Determination of mRNA levels of *Pcsk9* in the corresponding samples analyzed in panel A. Results show a representative blot (A) or the mean ± SD of the corresponding mRNA levels of 6 independent preparations, using *36b4* as internal control, and referencing hepatocyte content.

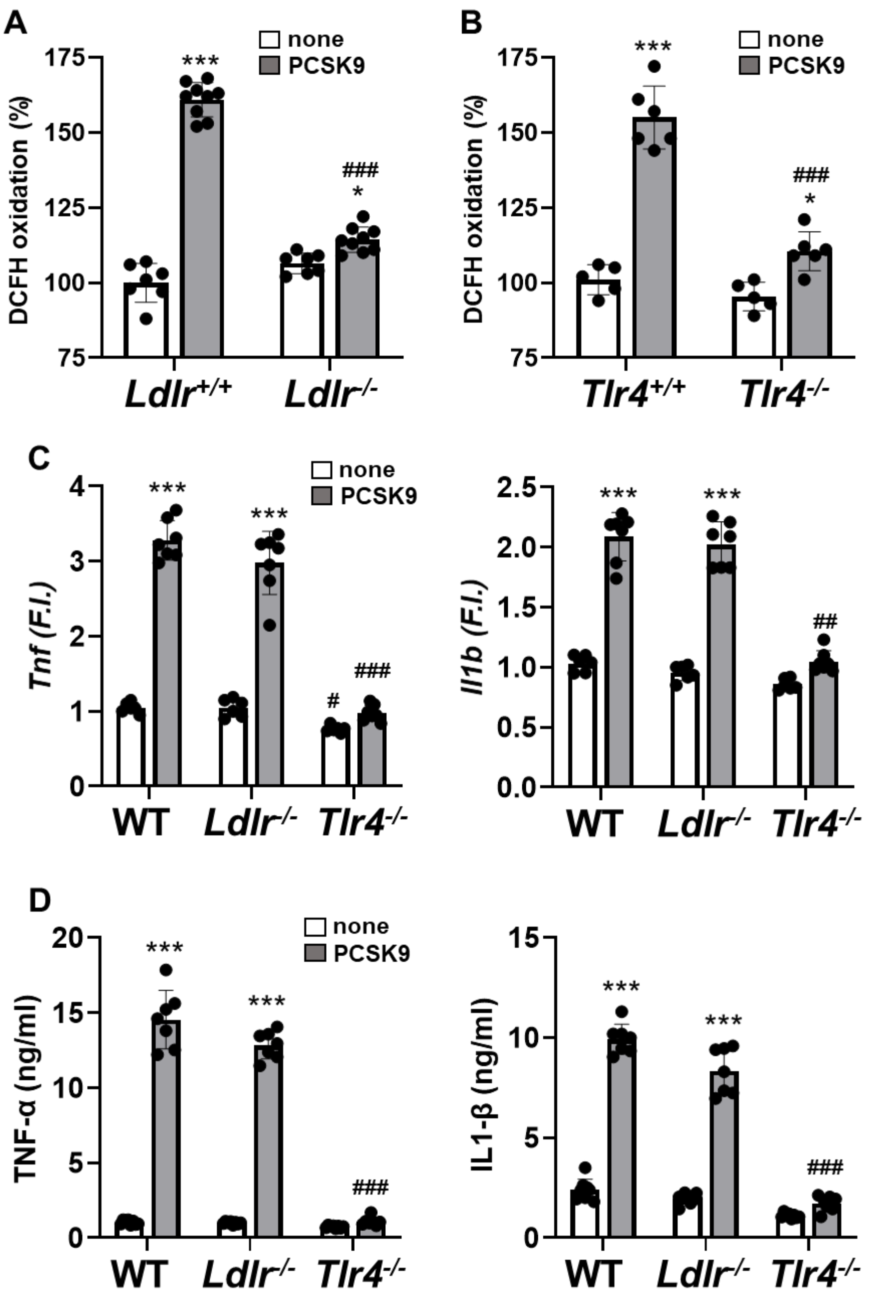

Figure S2. ***Effect of PCSK9 on the production of ROS and proinflammatory cytokines in elicited peritoneal macrophages from wild-type (WT), Ldlr*$^{-/-}$ *and Tlr4*$^{-/-}$ *mice.*** (**A-B**) Peritoneal macrophages from the indicated mice genotypes were treated for 18h with 1 μg/ml of recombinant PCSK9. The oxidation of DCFH was measured as an indication of ROS synthesis. (**C**) The mRNA levels of *Tnf* and *Il1b*, and (**D**) the release of TNF-α and IL-1β to the culture medium were determined in peritoneal macrophages from mice of the indicated genotype treated for 4h with 1 μg/ml of recombinant PCSK9. Data show the mean ± SD of macrophages from 5 mice of each genotype, assayed per triplicate. *P<0.05; ***P<0.001 *vs*. the corresponding condition in the absence of PCSK9. $^{\#}$P<0.05; $^{\#\#}$P<0.005; $^{\#\#\#}$P<0.001 *vs*. the corresponding WT counterparts.
